# A Conceptual Framework to Design Connected Mental Health Solutions in the United Arab Emirates: Questionnaire Study

**DOI:** 10.2196/27675

**Published:** 2022-02-07

**Authors:** Nidal Drissi, Sofia Ouhbi, Leena Amiri, Fadwa Al Mugaddam, Reem K Jan, Minna Isomursu

**Affiliations:** 1 Department of Computer Science & Software Engineering College of Information Technology United Arab Emirates University Abu Dhabi United Arab Emirates; 2 Department of Psychiatry and Behavioral Science College of Medicine and Health Sciences United Arab Emirates University Al Ain United Arab Emirates; 3 College of Medicine Mohammed Bin Rashid University Of Medicine and Health Sciences Dubai United Arab Emirates; 4 Faculty of Information Technology and Electrical Engineering University of Oulu Oulu Finland

**Keywords:** mental health, digital health, eHealth, connected health, mHealth, perceptions, attitudes, framework, design, UAE, mental health care professionals, Arab culture

## Abstract

**Background:**

Connected mental health (CMH) is a field presenting information and communications technology–based mental care interventions that could help overcome many mental care delivery barriers. Culture and background influence people’s attitudes, preferences, and acceptance of such solutions. Therefore, the suitability of CMH solutions to the targeted population is an important factor in their successful adoption.

**Objective:**

The aim of this study is to develop a framework for the design and creation of CMH solutions suitable for the UAE context. The framework is based on investigating enablers and barriers of CMH adoption in the United Arab Emirates, from the mental health professional's (MHP) perspective and from related literature.

**Methods:**

A survey of literature on relevant studies addressing the use of technology for mental care in Arab countries, and a web-based questionnaire-based survey with 17 MHPs practicing in the United Arab Emirates investigating their attitudes and views toward CMH was conducted. Results from the questionnaire and from related studies were analyzed to develop the design framework.

**Results:**

On the basis of findings from the literature survey and analyzing MHP answers to the web-based survey, a framework for the design of CMH solutions for the UAE population was developed. The framework presents four types of recommendation categories: favorable criteria, which included blended care, anonymity, and ease of use; cultural factors including availability in multiple languages, mainly Arabic and English, in addition to religious and cultural considerations; technical considerations, including good-quality communication, availability in formats compatible with mobile phones, and providing technical support; and users’ health and data safety considerations, including users’ suitability testing, confidentiality, and ensuring MHP integrity.

**Conclusions:**

CMH has the potential to help overcome many mental care barriers in the United Arab Emirates in particular and in the Arab world in general. CMH adoption in the United Arab Emirates has a potential for success. However, many factors should be taken into account, mainly cultural, religious, and linguistic aspects.

## Introduction

### Background

The connected mental health (CMH) field, referring to the use of information and communication technologies (ICTs) for mental health care, has become an established field of research, fueled by the continuous advances in technology and the existing barriers to mental care delivery. CMH includes different mental care solutions including, among others, mobile mental health, e–mental health, digital mental health, and telemental health, which have been increasingly investigated as alternatives or adjuncts to traditional care [1-3]. CMH solutions are available for several mental health conditions, including anxiety disorders [4-6], depression [7], and addiction [8]. CMH solutions are often associated with a number of benefits; they are convenient mental care delivery methods for the patient with regard to time and accessibility to treatment. CMH solutions can also help achieve a more widely spread delivery of mental care, reaching different groups of people. They can also address some of the limitations of the mental care systems, especially regarding costs and mental health professional (MHP) availability, which is currently further challenged by the COVID-19 pandemic. Moreover, CMH solutions can offer the treatment in a discreet and anonymous manner, which may help overcome the stigma barrier.

There is evidence on the effectiveness and acceptability of CMH solutions. Effectiveness has been previously demonstrated for interventions addressing anxiety and depression [9,10], as well as for issues related to exposure to traumatic events such as stress, insomnia, and substance abuse [11]. Acceptance and appropriateness of CMH solutions have been demonstrated globally by studies conducted in a number of countries including Germany [12], Australia [13,14], the Unites States [15], and Canada [16]. In general, positive attitudes around CMH solutions seem to stem from studies testing the outcomes of specific interventions and from participants completing the treatments [17]. However, several studies have revealed low acceptance and negative attitudes toward the use of CMH solutions, which were mainly reported to be related to preference of traditional in-person treatment [18,19], lack of prior awareness or use of CMH solutions [20,21], fear of exposure to false information, and access limitations [22].

MHPs play an important role in the success or failure of the adoption of CMH solutions with patients. With their help and inclusion, effective solutions could be co-designed, and weak aspects of mental care delivery could be identified and addressed. However, MHP attitudes toward CMH solutions are mixed and depend on many factors, including patients’ characteristics, types of CMH solutions, and adoption conditions. Several studies have investigated MHP attitudes toward CMH solutions; examples of such studies include, among others, a study conducted in England, which showed overall positive MHP attitudes toward digital solutions. The study showed that younger MHPs are more open to using CMH solutions. However, it also identified limitations to adoption, including that some MHPs reported lack of confidence using technology and expressed concerns regarding security and confidentiality [23]. A study conducted in Australia also showed positive MHP attitudes and reported their concerns regarding patients’ accessibility to CMH solutions and their ability to use them [24]. Similar concerns regarding patients were also reported in a study conducted in the United States [15]. In addition, a study conducted in the United Kingdom reported MHP preference of guided use of CMH solutions by the MHP rather than independent use by the patient [25].

Culture and cohort characteristics are also important factors that significantly influence CMH adoption. Although people from different cultures may share some characteristics [26] and preferences regarding certain ICT tools [27], a standard unified approach of ICT intervention design for all cultures might not be successful [27]. Culture is an important factor, as it influences attitudes and preferences [28]. Culture has been found to have an influence on users’ preferences and attitudes toward different ICT-based interventions [29-31]. Examples include a study investigating important factors to take into account when designing websites in different cultures (British and Omani), revealing that the users might have similar preferences; however, there were significant differences in the importance and priorities of the preferences. Another study investigated the influence of culture on computer interface acceptance by comparing interface preferences between a group of Australian students and a group of international students, has reported that culture not only influenced the design preferences but also influenced the acceptance, attitudes, and behavior [32].

### Objective

There is a scarcity of data on CMH use from the Arab region, even though many of the Arab countries struggle with barriers to mental care delivery [33-35] that could be solved or moderated with the adoption of CMH interventions. To address the paucity of CMH-related research in Arab countries, and to gain insight into the possibility of adoption of CMH in an Arab environment, this study focuses on CMH use in the context of the United Arab Emirates. The Arab world includes the areas known as the Middle East and North Africa. Arab countries can be defined as those where Arabic is the dominant language; they are also religiously and ethnically diverse, with Islam being the dominant religion in most countries [36]. The United Arab Emirates is an Arab country, with distinctive characteristics that include significant numbers of expatriates from different nationalities, which makes it home for different cultures. It must be noted that although different Arab cultures are usually referred to as a unified *Arab culture*, each Arab country has its own unique specifications and characteristics [37]. Therefore, investigating the specific needs and properties of the targeted population is important for the success of any intervention.

This study proposes a framework to design CMH interventions for the UAE population by analyzing related publications and MHP attitudes and views toward the use of CMH interventions. The study identifies facilitators, barriers, and strengths of CMH adoption and extracts necessary elements for the success of CMH adoption in the United Arab Emirates. The framework could also be used in the design of CMH solutions for other Arab countries or populations. Given that design preferences influence the acceptance and behavior [32], the development of suitable design frameworks for the United Arab Emirates and Arab countries might help increase users’ acceptability of this type of mental care intervention and promote CMH adoption.

## Methods

### Research Design

This study aims to develop a framework to design CMH solutions for the United Arab Emirates. To achieve this objective, a 2-step investigation method was followed. The first part consisted of a literature survey of related studies conducted in Arab countries, to extract relevant findings regarding the advantages and barriers of CMH adoption, in addition to recommendations and guidelines on CMH use. The second part investigated the perceptions and attitudes of MHPs in the United Arab Emirates toward the use of digital technologies for mental health care as well as enablers and barriers of CMH adoption. A survey was carried out using a web-based questionnaire delivered to a group of MHPs practicing in the United Arab Emirates. The questions were designed in collaboration with 2 MHPs and investigated MHP knowledge on CMH, their previous use and willingness to use CMH solutions, best modalities of CMH adoption, as well as their concerns regarding use of CMH. The multiple-choice answers included options that had a significance to the UAE context and culture.

### Literature Survey

To construct an understanding of studies addressing the use of technology for mental health in Arab countries, we have conducted a survey of literature following the PRISMA (Preferred Reporting Items for Systematic Reviews and Meta-Analyses) protocol [38] in the databases Scopus and Google Scholar using search strings based on the following keywords: *Arab*, *Countries*, *Culture*, *Attitudes*, *Acceptance*, *Use*, *Connected mental health*, *Digital mental health*, *e-mental health*, *Mobile mental health*, and *Tele-mental health*.

The selected studies were investigated and analyzed to extract three main categories of information: advantages of CMH adoption, barriers to CMH adoption, and recommendations for successful CMH adoption.

### Web-Based Survey

#### Data Collection

A self-administered web-based questionnaire, created using Google Forms, was shared with MHPs in the United Arab Emirates via emails. The mailing list to target was provided by MHPs collaborating as coauthors in the study, which contained emails of MHPs from different health institutions in the United Arab Emirates. The questionnaire was pretested by the authors before sending it to the MHPs. The estimated time to complete the questionnaire was 10 minutes, and the answers were collected anonymously, which was stated in the questionnaire. Permission was obtained from the relevant authorities at the UAE University. Information provided about the questionnaire is based on the Checklist for Reporting Results of Internet E-Surveys [39].

#### Survey Questions

The web-based questionnaire included 18 questions, 13 main questions, and 5 subquestions depending on the answers selected, as presented in Table 1. The first 2 questions were basic questions to provide demographic data (job title and gender). Question Q3 investigated knowledge of MHPs on terms used in the literature to refer to the use of ICT for mental care. Q4-Q9 investigated MHP opinions on different aspects of use of CMH, including cases where CMH could be helpful, whether CMH could assist or replace traditional care, whether CMH could be adopted in the United Arab Emirates, and what elements could promote and improve its adoption. Q10 and Q11 investigated previous MHP use of CMH with their patients, the latest experience with CMH, and MHP willingness to use or reuse CMH with their patients in the future. Q12 and Q13 investigated MHP concerns regarding CMH use and elements that should exist in CMH solutions. Table 1 presents the questions, objectives, and types of answers.

**Table 1:** Survey questions.

ID and objective				Question		Type of answer
**Providing basic information on the participants**						
	Q1		Job title		Open answer	
	Q2		Gender		Multiple choice	
**Investigating MHP^a^ knowledge of the terms used to refer to the use of ICT^b^ for mental health care, as well as investigating their opinion on different aspects of using digital solutions for mental health care **						
	Q3		Do you know the following terms? (e-mental health, mobile mental health, connected mental health, digital mental health, telemental health)		Yes or no (for each term)	
	Q4		In your opinion, in which case could the use of digital technology for mental health care be helpful?		Multiple choice	
	**Q5**		Do you think that digital technology for mental health can assist psychiatric therapy?		Yes, no, I don’t know or in some cases	
		Q5.1	If you answered “In some cases,” please provide examples of cases		Open answer	
	**Q6**		Do you think that therapies delivered via digital technology can replace those delivered face-to-face?		Yes, no, I don’t know or in some cases	
		Q6.1	If you answered “In some cases,” please provide examples of cases		Open answer	
	Q7		In your opinion, what could be the barriers to seeking mental care in the United Arab Emirates that could promote the use of digital solutions?		Multiple choice	
	**Q8**		Do you think digital solutions for mental care can be adopted in the United Arab Emirates?		Yes, no, or I don’t know	
		Q8.1	If you answered “No,” please explain why		Open answer	
	Q9		In your opinion, what of the following can improve the adoption of digital solutions for mental health in the UAE culture?		Multiple choice	
**Investigating MHP previous use of digital solutions with their patients, and their willingness to use them in the future**						
	**Q10**		Have you ever used a digital solution with your patients?		Yes or no	
		Q10.1	If you answered “Yes,” how were your patients’ attitudes toward it?		Open answer	
	**Q11**		Would you be willing to use a digital solution with your patients in the future?		Yes, no, or I don’t know	
		Q11.1	If you answered “No,” please explain why		Open answer	
**Identifying MHP concerns regarding the use of digital solutions for mental health care and their recommended features to be implemented in such solutions**						
	Q12		What concerns do you have regarding the use of digital solutions for mental care by patients?		Open answer	
	Q13		In your opinion, what are the critical elements and features that should exist in digital health solutions for mental care?		Open answer	

^a^MHP: mental health professional.

^b^ICT: information and communications technology.

## Results

### Literature Survey Results

#### Selection Results

A total of 6 relevant studies were selected. The studies have addressed different Arab countries, including Lebanon, Egypt, and Saudi Arabia, and have put forward several significant findings as presented in the following subsections. The studies’ selection as well as information of the countries addressed and the aims of the studies are presented in Table 2.

**Table 2:** Selection results.

Reference	Country or cohort	Aim
Kamel et al [34]	Egypt	Understanding the opinions of psychiatrists on the state of mental health care services in Egypt, their attitudes toward web-based interventions and telemedicine for mental health, and their current knowledge and perceived advantages regarding electronic mental health
Ashfaq et al [40]	Syrian refugees and other vulnerable Arab populations	Evaluating available literature on the use acceptability of mobile mental health in Syrian refugees and other vulnerable Arab populations
Harper Shehadeh et al [41]	Lebanon	Presenting preliminary findings on the feasibility of a minimally guided World Health Organization e-mental health intervention in Lebanon
Abi Ramia et al [33]	Lebanon	Informing the cultural adaptation of an internet-delivered mental health intervention in Lebanon based on a multi-stakeholder perspective
Knaevelsrud et al [42]	War-traumatized Arab patients, focusing on Iraq	Investigating the efficacy of a cognitive behavioral internet-based intervention for war-traumatized Arab patients, with a focus on Iraq
Binhadyan et al [35]	Saudi Arabia	Assisting mental health services in Saudi Arabia by focusing on e-mental health and introducing possibilities and challenges in transforming the e-mental health services of Australia to the Saudi Arabian health care context

#### Main Findings

#### Advantages of CMH Adoption

Few studies have investigated CMH adoption in Arab populations, revealing different possible advantages, including reduction of financial, physical, and societal barriers to mental care [33-35], overcoming access limitations related to location and time [35], provision of anonymous access to care [34], reduction of the stigma associated with traditional care [33-35], lowering mental care systems’ accessibility threshold, improving therapists’ efficacy, provision of psychoeducation [34,35], and wider mental care coverage, as well as reaching vulnerable people [40] and people in remote areas [34].

#### Barriers to CMH Adoption

Even though CMH might bring solutions to several mental care issues in Arab countries, certain barriers might obviate its adoption. Existing barriers include lack of awareness of the potential seriousness of mental issues [33]; prioritizing other life needs over mental care [33]; high level of illiteracy [33,34,41]; electronic illiteracy, especially among older adults [33,35,40]; concerns regarding confidentiality, privacy, and security [33,34]; resistance to change [34]; lack of content in Arabic [34]; cultural incompatibility [34,40]; technological problems [34]; beliefs that CMH interventions would negatively affect the rapport between patients and MHPs [34]; inadequate technological access, especially for people in conflict regions [40,42]; poor CMH credibility [40]; preference for traditional care [40]; lack of knowledge about CMH [41]; and lack of trust in CMH [42].

Wide use of mobile phones and the wide internet coverage were reported as facilitators to CMH adoption [33,40]. However, several barriers to technology use were reported as well; for example, lack of access and difficulties connecting to the internet [33,40], slow and low quality of internet connection [33], and costs of mobile and internet access [40]. Stigmatization was among the barriers reported to CMH adoption, owing to the stigma of admitting the existence of mental issues and the associated fear of rejection even by family or society [40,42].

#### Recommendations for Successful CMH Adoption

To overcome some of the barriers to mental care delivery and to CMH adoption in the Arab world, some studies put forward some recommendations. These included ensuring access to good-quality internet connection [41] and making the interventions available in formats compatible with mobile phones [33]. CMH interventions should take into consideration the users’ time availability and lifestyle [33] as well as demographics, employment status, family status, previous experiences, and traumas such as war or losing loved ones [33]. When catering to the Arab world, differences between genders in the culture should be considered to make the solutions more relatable; however, unhelpful gender stereotypes should be avoided [33,35]. It is also important to investigate and focus on the mental care needs specific to the targeted population and to provide compatible solutions with those needs [34,40].

To overcome trust and credibility issues as well as the preference of traditional care barrier, blended care could be a helpful approach, in addition to promoting the interventions through trusted parties [33,34]. CMH interventions for Arab populations should incorporate linguistic, cultural, social, and religious considerations [33,35,40].

### Web-Based Survey of MHPs

#### Demographics of the Web-Based Survey Participants

A total of 17 MHPs participated in the survey, and the majority of participants were females (12/17, 71%). The participants occupied different psychiatric posts including consultant psychiatrists, faculty members in psychiatry, medical research specialists, and psychiatrists, who all could be referred to as *experts*, as well as psychiatry residents, who constitute the majority of the respondents (5/17, 29%). Table 3 summarizes the demographic characteristics of the participants.

**Table 3:** Demographic of participants (N=17).

Variables			Participants, n (%)
**Gender**			
	Female	12 (71)	
	Male	5 (29)	
**Job titles**			
	Consultant psychiatrist	5 (29)	
	Faculty member in psychiatry	3 (18)	
	Medical research specialist	1 (6)	
	Psychiatrist	3 (18)	
	Psychiatry resident	5 (29)	

#### Survey Results

#### Q3: MHP Awareness of the Terms e-Mental Health, Mobile Mental Health, CMH, Digital Mental Health, and Telemental Health

**Figure 1:**
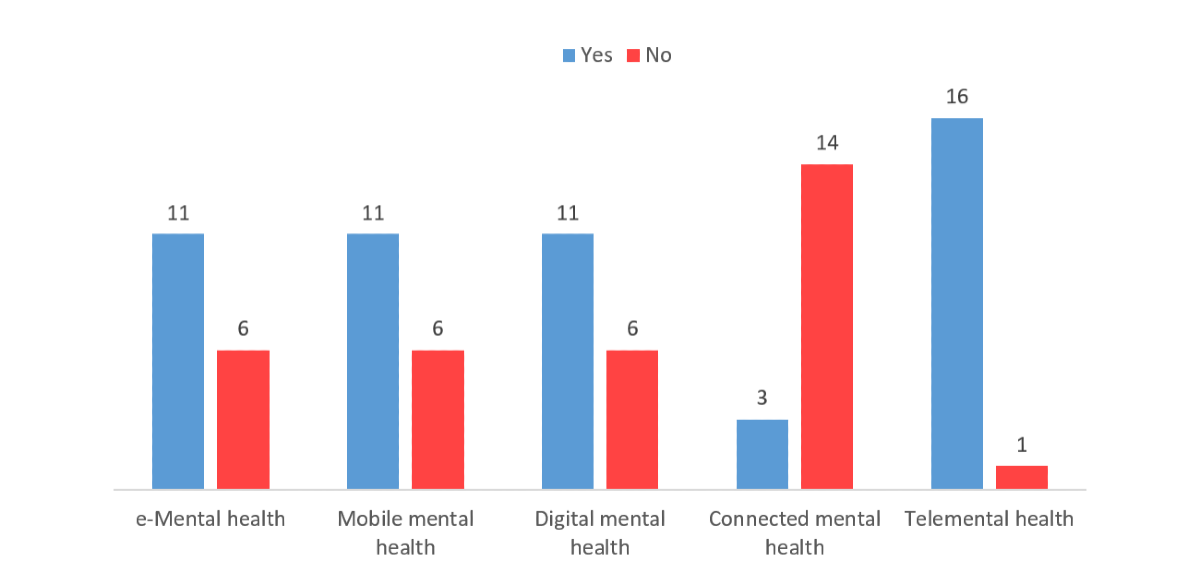
Mental health care professionals’ knowledge on the terms referring to the use of information and communication technologies for mental care.

When investigating knowledge of MHPs on the different terms referring to the use of technology for mental health, *telemental health* was found to be the most known term, as only 6% (1/17) of the participants did not recognize it, whereas *CMH* was the least known term, as 82% (14/17) of the participants did not recognize it. The rest of the terms were found to be equally recognized, with 65% (11/17) of the participants recognizing each term. Figure 1 summarizes the answers to this question.

#### Q4, Q5, and Q6: Cases and Modalities of CMH Use

Q4 investigated in which cases digital technology could be helpful in mental health care. Answers to this question included common mental problems (depression, stress, anxiety, etc), serious mental problems (schizophrenia, bipolar disorder, dementia, etc), none, and other. Almost all respondents (16/17, 94%) reported that digital solutions could be beneficial in case of common mental disorders, whereas 24% (4/17) of them indicated that it could be beneficial for both common and serious mental disorders, and 6% (1/17) of the participants reported not having an idea of best cases for use.

**Figure 2:**
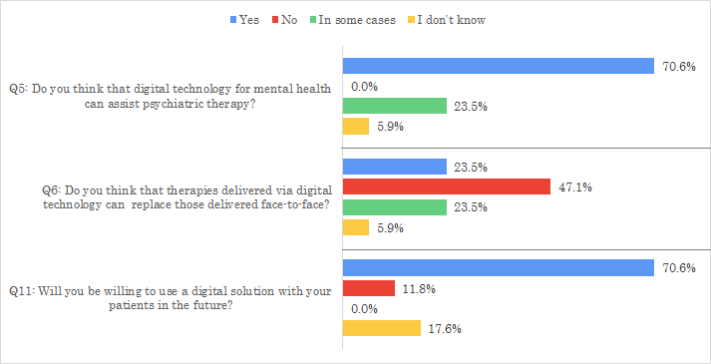
Answers to questions Q5, Q6, and Q11.

Q5 investigated, based on MHP opinions, whether digital solutions can assist psychiatric therapy. The majority of the participants (12/17, 71%) answered “Yes,” 24% (4/17) of the participants answered “in some cases,” and 6% (1/17) of the participants answered “I don’t know.” Figure 2 summarizes the answers to this question. When comparing answers of experts in the sample with those of residents, no major differences were identified for this question, as both reported answers that varied between “Yes” and “In some cases,” and none of the participants reported that digital solutions could not assist in psychiatric therapy.

Participants who answered “In some cases” were asked to provide examples of such cases in Q5.1. Their answers are listed in Textbox 1.

Q6 investigated whether digital solutions could replace traditional face-to-face methods of mental care delivery. Overall, 47% (8/17) of the participants answered “No,” whereas equal proportions (4/17, 24%) responded “Yes” and “In some cases,” as presented in Figure 2. When comparing answers of experts with those of residents for this question, residents seem to be more open to the possibility of CMH replacing traditional therapy, as 58% (7/12) of experts and 20% (1/5) of residents answered “No.”

Participants who answered “In some cases” were asked in Q6.1 to provide examples of such cases. Their answers are presented in Textbox 2.

**Cases where digital solutions could assist psychiatric therapy.**

**Participant quotes**

- “Some disorders can be managed through digital technology, such as anxiety disorders that does not require medication, application of cognitive behavioral therapy” [P5]
- “Patients who have low risk to harm them or to other, have social support, have normal IQ” [P8]
- “Anxiety; Some cases of depression; insightful schizophrenic patient and bipolar; poor insight schizophrenic and bipolar patients with strong family support; stable dementia patient with family support” [P13]
- “Mild cases with low risk” [P14]

**Cases where digital solutions could replace the traditional face-to-face methods.**

**Participant quotes**

- “I believe most cases requiring cognitive behavioral therapy can be done entirely through digital technology” [P5]
- “Cases with low risk to harm themselves or others; patients with social support; patients that have normal IQ” [P8]
- “Anxiety; some cases of depression; insightful schizophrenic patient and bipolar; poor insight schizophrenic and bipolar patients with strong family support; stable dementia patient with family support” [P13]
- “People with a certain intellectual capacity can engage, however those on the spectrum or intellectually disabled or other neuro-cognitive issues such as dementia or traumatic brain injury (TBI) etc, need in person assistance. Digital health should be an adjunct to human therapy not a replacement” [P17]

#### Q7: Barriers to Mental Care Seeking in the United Arab Emirates

Q7 was a multiple-choice question. The answers’ options for Q7 included cost, stigma, shortage in MHPs, distance from MHPs, lack of knowledge on mental health, and other. The majority of participants (13/17, 76%) reported that the most probable barrier to mental care delivery in the United Arab Emirates was stigma, followed by distance from MHPs, and shortage in MHPs. Table 4 presents the answers to Q7.

**Table 4:** Barriers to mental care delivery in the United Arab Emirates based on mental health professional (MHP) views (N=17).

Mental care delivery barriers	Participants, n (%)
Stigma	13 (76)
Distance from MHPs	11 (65)
Shortage in MHPs	10 (59)
Cost	8 (47)
Lack of knowledge on mental health	7 (41)

#### Q8 and Q9: Adoption of CMH Solutions in the United Arab Emirates and Factors That Could Help Their Adoption

Q8 investigated whether digital solutions for mental care can be adopted in the United Arab Emirates based on the MHP opinion. This question can be answered by “Yes,” “No,” or “I don’t know.” Of 17 participants, 16 (94%) answered “Yes” to this question, whereas only 1 (6%) participant, who was an expert, answered “No.” Participant (P14) who answered “No” justified their choice with the following statement:

Digital solutions are unlikely to be able to replace face to face interactions as observation of mental state is such a vital part of our assessment. As mental state is dynamic, digital solutions provide little opportunity to access it repeatedly.

Q9 investigated which elements can be incorporated in digital solutions for mental care to promote their use in the United Arab Emirates. It is a multiple-choice question with the following answer choices: digital solutions developed in the United Arab Emirates; availability in Arabic and English; religious content, such as Ayat from the Quran or Adkar; or other. The majority reported that availability in Arabic and English (14/17, 82%), as well as being developed in the United Arab Emirates (13/17, 76%), could help promote the use of digital solutions for mental care in the United Arab Emirates. In addition, 1 participant also reported the necessity to consider the different cultures in the UAE population by the following statement: “catering for Urdu/Hindi/Tamal/Arabic/English, at least.” Table 5 presents the answers to Q9.

**Table 5:** Factors that could help the adoption of connected mental health in the United Arab Emirates (N=17).

Factors	Participants, n (%)
Availability in Arabic and English	14 (82)
Digital solutions developed in the United Arab Emirates	13 (76)
Religious content, such as Ayat from the Quran or Adkar	9 (53)

#### Q10 and Q11: MHP Previous Use of CMH Solutions With Their Patients and Their Willingness to Use Them in the Future

In response to Q10, 53% (9/17) of the participants reported previous use of digital solutions, representing 50% (6/12) of experts and 60% (3/5) of residents. These participants were asked about their patients’ attitudes toward the use of digital solutions in their treatment. Their answers are presented in Textbox 3.

Q11 investigated the willingness of MHPs to use digital solutions for mental care in the future. The majority of MHPs (12/17, 71%) answered “Yes,” reporting their willingness to use digital solutions in the future, whereas 18% (3/17) of the MHPs answered “I don’t know,” 2 (67%) of whom were residents and 1 (33%) expert. Figure 2 summarizes the answers to this question.

Only 2 MHP, who were *experts*, answered “No” to Q11, of whom 1 (50%; P14) provided a reason for their answer as follows:

Not on a long-term basis. I think it will affect the quality of assessment and also will affect the patient doctor relationship, which is so important in psychiatry.

**Patients’ attitudes toward the use of digital solutions in their treatments.**

**Participant quotes**

- “Generally positive” [P2]
- “Acceptable” [P4 and P6]
- “Happy about it, and some of them were cooperative” [P8]
- “Satisfied” [P12]
- “With serious mental health disorders, it was difficult to reach some poor insight patients, with stable patients and insightful it was welcomed” [P13]
- “Not everyone finds it useful. Some are okay with it as a short-term solution” [P14]
- “Appreciated” [P16]
- “Depends on the patient. Some like it and maximize its benefit, some can barely follow through” [P17]

#### Q12: MHP Concerns Regarding the Use of Digital Solutions for Mental Care

The participants provided a list of concerns, including, among others, concerns regarding the importance of in-person and physical examinations, security and confidentiality concerns, and patients’ suitability concerns. The reported concerns are presented in Textbox 4.

**Reported concerns regarding the use of digital solutions for mental care.**

**Participant quotes**

- “The community response towards change in treatment therapy; Affordability (insurance coverage for some categories of the community); The adaptation of digital solutions into the cultural, behavioral, and language aspects of the country” [P1]
- “Lack of human touch” [P2]
- “Need for physical examination” [P4]
- “Missing high risk patients, inability to reach them in time; Confidentiality; Adherence of patient” [P5]
- “Assessment is jeopardized in many of the psychiatric conditions such as psychosis; Hard to implement it with demented patients unless already diagnosed and the contact is with the family member” [P6]
- “I need face to face assessment as so many symptoms could be reveal only by us” [P7]
- “Need for face to face evaluation and assessment for reaching a plan” [P8]
- “The geriatric patient will need assistance, people might use it to play doctor on others” [P10]
- “Impaired rapport” [P11]
- “Sometimes you need to see the patient face to face to exclude medical causes” [P12]
- “Some Patients are unable to provided the comfortable space that they can find by meeting the doctor face to face, that can easily released their emotions adequately, the signs of relapses can be missed if no strong family support; The compliance to medications and Follow-up (FU), the Electrocardiogram (ECG), lab investigators that done regularly to patients under psychotropic medication” [P13]
- “Lack of frequent observation and proper assessment” [P14]
- “Shouldn’t replace the psychiatrist or therapist, medico-legal and ethical concerns” [P15]
- “Technical support regarding opening files, documentation and insurance coverage” [P16]
- “Over utilization and relying solely on digital input, not utilizing or building on coping skills. Social isolation” [P17]

#### Q13: Critical Elements and Features That Should Exist in Digital Health Solutions for Mental Care

Participants provided a list of critical features that they believe should be incorporated into CMH solutions for mental care. Provided elements included, among others, cultural and linguistic adaptation, patients’ suitability testing, ensuring communication between MHPs and patients, and risk or crisis management features. The participants’ recommended features are presented in Textbox 5.

**Features and elements that should exist in connected mental health solutions.**

**Participant quotes**

- “Adaptation of language and cultural aspects; Affordability; Good Advocacy and promotion for digital Health Methods” [P1]
- “Digital health solutions should supplement the traditional practice rather than replacing that” [P2]
- “Mix of digital and face to face contact” [P4]
- “Confidentiality, culturally sensitive, uses both English and Arabic, is able to rule out high risk patients” [P5]
- “Camera” [P6]
- “Missing important point in assessment, difficult to reach to some people, at times need to reach to the relative but was difficult” [P8]
- “Easy use and understandable instructions and information for the patients” [P10]
- “Video conferences should be an option” [P11]
- “Video” [P12]
- “Video camera from patients side, the speed of WiFi” [P13]
- “It should be user friendly, dynamic and approachable by vast majority. There should be means of auditing the use of digital health pros and cons. Training for professionals is essential” [P14]
- “Unified regulations” [P15]
- “Confidentiality” [P16]
- “For me the most critical entity that is lacking severely and can be built through Digital health for Mentally ill is a platform to connect patients to resources. In a society with limited resources for the lonely and isolated depressed individuals, these platforms should create network and decrease the isolation” [P17]

## Discussion

### Principal Findings

Awareness of MHPs in the United Arab Emirates of the terms used to refer to the use of ICT for mental care solutions is aligned with the changes in this research field. Almost all surveyed MHPs had knowledge of the term *telemental health*, which is related to the term *telehealth*, one of the oldest known terms referring to the use of ICT in health, as it has been used in the literature since the 1990s [43,44]. The terms *e-mental health*, *mobile mental health*, and *digital mental health*, are also known terms that have been used in the literature, especially in more recent years [1], which was mirrored in the participants’ answers, as the majority reported knowledge of those terms. The majority of MHPs were unaware of the term *connected mental health*; this finding is in line with the changes in the literature, as even though *connected health* is an established field [45], the term *connected mental health* is rarely used in the literature [1].

Surveyed MHPs acknowledged the benefits of CMH, as the majority believed that CMH could assist in psychiatric therapy. However, it must be noted that the majority of participants, especially experts, reported that CMH solutions could not be a replacement for traditional therapy but should be an adjunct to traditional care. Openness of resident participants to the idea of CMH replacing traditional therapy might be explained by them generally being from the young generation, who are familiar with the use of ICT tools, which might have resulted in them having more trust in CMH than experts. Combining traditional and CMH-based therapies or including MHPs in the CMH solutions could be beneficial in promoting and adopting CMH interventions in the United Arab Emirates and in Arab countries in general, as physicians in the Arab culture are much respected and trusted [33,46]. Involvement of MHPs in the CMH solutions may present additional benefits in the UAE context. It may increase the credibility of the treatment and hence adherence to it and may help overcome the barrier of preference of traditional care. Blended care and inclusion of MHPs could also ensure suitability of patients to use CMH solutions. The majority of surveyed MHPs reported that CMH could mainly be used with people with common mental issues, patients with normal IQ and normal cognitive skills, and those who would not present any harm to themselves or to others.

Arab countries face many barriers to mental care seeking and delivery. Such barriers include financial problems, unemployment [33], poorly equipped mental care systems [34], stigma, therapists’ availability, as well as cultural barriers such as faith healing beliefs and sex segregation [35]. On the basis of our results, in the case of the United Arab Emirates, stigma followed by MHP availability were the most reported barriers to mental care access. Stigma is one of the barriers most often associated with mental health issues. The suffering of people with mental disorders is not limited to their mental issues’ symptoms, which include distress and disability that keep them from living a normal life and achieving their goals; their suffering is extended by the stigma related to the mental issues, causing them to experience social injustice, discrimination, misinterpretation of their mental state, and stereotyping [47,48]. Stigma might be one of the major barriers to mental care seeking in the Arab countries, as in the Arab culture, seeking help from MHPs is viewed as a sign of weakness and a shameful act that impacts not only the individual but also his or her family [37]. The Arab culture is more family or tribe and community oriented and not individual oriented; decisions are usually taken on a collective level rather than on an individual level to best serve the collective interest [46].

Adoption of CMH interventions could mitigate issues related to stigma and community concerns by providing anonymous and discreet access to care. This, however, does not imply that stigmatization against people with mental issues is normal; it provides a possible solution to overcome it and receive the needed care, as changing stigmatization attitudes have been proven to be a difficult challenge, and implemented strategies to face it were found to be ineffective [47]. On the basis of the results, the availability of MHPs is another barrier to seeking mental care in the United Arab Emirates. However, this barrier could as well be mitigated with the adoption of CMH. CMH interventions could ensure access to care for people in isolated or remote areas and reduce the load on mental care systems, especially considering the wide coverage of internet access and technology use in the United Arab Emirates.

When asked if CMH interventions could be adopted in the United Arab Emirates, the majority of the participants confirmed its possibility and provided several factors that could boost its adoption. The reported factors were mainly related to cultural and linguistic considerations. Lack of consideration of those factors was reported as a barrier to CMH adoption in studies addressing other Arab countries [34,40]. Inclusion of religious content was also believed to be an important factor by more than half of the participants. Religion plays an important role in the Arab culture; it influences the individual’s beliefs, life, and behavior [46]. In addition, many Arabs rely on religion for psychological symptom formation, attribution, and management [46]; therefore, its inclusion could make the user more comfortable, accepting, and trusting toward CMH interventions.

As every country or population has its own characteristics, beliefs, and struggles, cultural, economical, and religious considerations may be necessary factors for the successful adoption of CMH in the Arab countries, including the United Arab Emirates. However, it must be noted that in the case of the United Arab Emirates, the majority of the citizens are expatriate residents from different countries with different backgrounds, which should not be neglected when creating interventions for the general population. This insight was also expressed by 1 of the participants who proposed to cater to the different cultures in the United Arab Emirates as a factor that could boost the CMH adoption in the United Arab Emirates. Only 1 participant expressed unacceptability regarding the CMH adoption in the United Arab Emirates, with concerns mainly around the importance of continuous and face-to-face observation of the mental status of the patients and the possibility that CMH might limit or jeopardize these aspects. Similar findings were reported in a study investigating views of Egyptian psychologists on e-mental health, who reported that it might negatively affect the rapport between patients and MHPs [34]. These concerns further confirm the importance of the inclusion of MHPs in the design and creation of CMH solutions.

CMH solutions were moderately used by the surveyed MHPs with their patients; approximately 53% (9/17) reported previous use of such solutions, which is in line with the general slow and limited adoption of CMH solutions in the clinical context [49]. Investigated MHPs who have previously used CMH solutions with their patients generally reported positive results. Although some of the participants reported unfavorable outcomes, these were mainly related to patients’ characteristics and suitability, which emphasizes the need for users’ suitability assessment when using CMH solutions. The majority of participants, including those who have never used CMH interventions with their patients, were open to using them in the future; 2 participants were not open to using CMH, 1 of whom expressed concerns regarding the importance of the physician–patient relationship and the quality of treatment, especially in the long term. Throughout their answers, MHPs repeatedly expressed concerns regarding the importance of MHP input and management of the patients, which again implies that blended care might be the most suitable approach for CMH interventions to be adopted in the United Arab Emirates.

### A Conceptual Framework to Design CMH Interventions for the UAE Context

#### Overview

**Figure 3:**
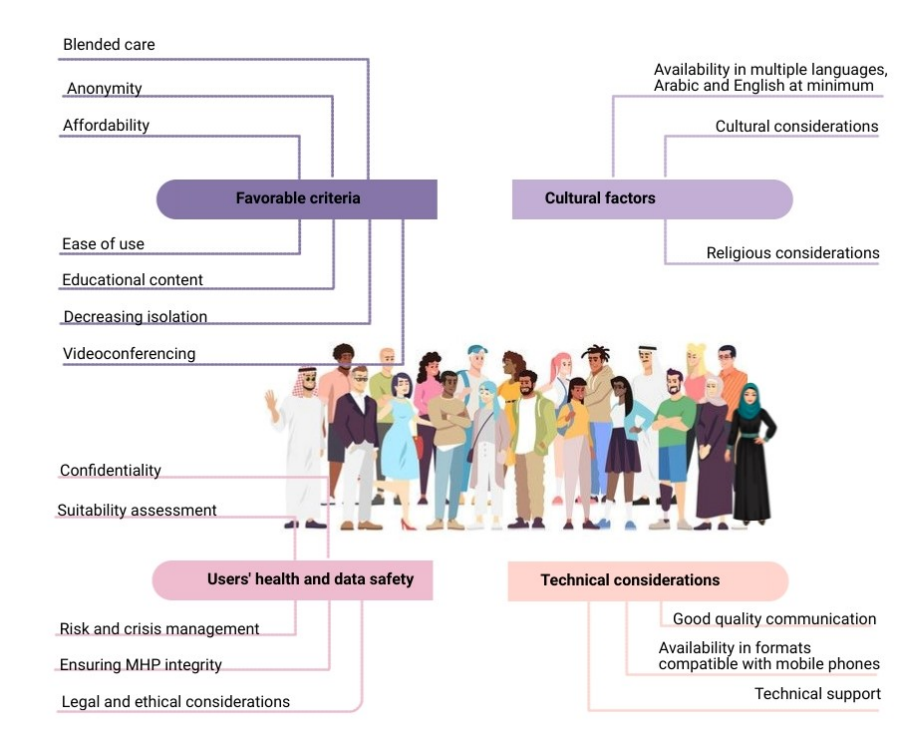
Factors to consider when designing connected mental health interventions for the UAE population. MHP: mental health professional.

Surveyed MHPs were asked to provide their concerns regarding the adoption of CMH interventions as well as critical elements and features that they think should exist in CMH solutions. On the basis of the list of concerns and critical elements as well as the answers of participants to the entire survey, a framework for the design of CMH solutions for the UAE population, including Emiratis and expatriates, with a focus on people of an Arab nationality was developed. The framework was also reviewed and validated by 2 MHPs collaborating in the study. The main points of this framework are presented in Figure 3.

#### Favorable Criteria

#### Blended Care

*Blended care* may be a trusted approach that has a better chance of success in the United Arab Emirates than independent CMH solutions. Blended care can provide the benefits of technology without losing those of traditional care and of communicating with MHPs. In addition, MHP inclusion and presence in CMH solutions could increase the patients’ trust in the solutions and promote their use.

#### Anonymity

Anonymity could help encourage people to use CMH solutions to seek the needed care, overcoming stigma barriers related to mental health.

#### Affordability

Costs of professional mental care in the United Arab Emirates are relatively high, which constitutes a barrier to mental care seeking, especially for non-Emirati expatriates. The cost issue is further aggravated by either limited or nonexistent coverage of mental care from insurance companies, as the majority of insurance plans do not include mental care coverage [50,51]. CMH interventions should help overcome the cost barrier by offering free and more affordable solutions. In addition, providing insurance coverage for the use of paid CMH solutions could mitigate the affordability issue.

#### Ease of Use

CMH solutions should be simple and easy to use and understand for the patients to be comfortable using them.

#### Providing Educational Content on Mental Health

There is a lack of knowledge on mental health and its importance. CMH solutions should provide educational content that is easy to access and understand, which could help spread awareness on the importance and seriousness of mental health.

#### Providing Recommendations and Advice That Decrease Isolation

One of the concerns regarding the use of CMH interventions is decreasing the user’s social interaction, which is usually already impaired in people with mental issues. Therefore, CMH solutions should include tips and recommendations that would encourage the user to have social interactions with their families, friends, and community, which would decrease their isolation. Examples of such recommendations include proposing outdoor activities in addition to tasks that incorporate family or friends.

#### Videoconferencing

Videoconferencing is one of the communication modalities that could be integrated in blended care, which could provide face-to-face communication without the need to be in the same location as the MHP. Face-to-face communication could be imperative for certain cases. In addition, MHPs could extract important information from physical observation of the patients, including their gestures, movements, and facial expressions. However, certain points should be respected when adopting blended care in the United Arab Emirates through solutions such as videoconferencing, with the major one being accommodation for Hijabi women. Most Emirati women wear Hijab, which requires them to dress in a certain way in public and when around males who are not related to them, so is the case for many Arab and Muslim women. There are also women who wear Niqab, which requires them to cover their faces. Therefore, when communicating with an MHP especially via videoconferencing, women should be informed beforehand of the gender of the MHP, so they could be prepared and not to cause them any discomfort [52]. In addition, they should preferably have the ability to choose the MHP gender. Both men and women in the Arab culture might not feel comfortable discussing certain sensitive subjects. Therefore, CMH solutions for the United Arab Emirates and for Arab countries in general should provide a gender choice. Not respecting limitations and habits regarding interactions between the genders might present a major barrier to CMH adoption in the United Arab Emirates.

#### Cultural Factors

#### Availability in Multiple Languages, At Least in Arabic and English

Providing CMH solutions in the native languages of all residents of the United Arab Emirates might not be feasible. Therefore, CMH interventions should at least be provided in Arabic for Emirati and Arab expatriates and in English for non-Arab expatriates. Arabic and English are the 2 most spoken languages in the United Arab Emirates, and availability in these 2 languages is important to reach a large proportion of the United Arab Emirates's population.

#### Cultural Considerations

The Arab culture has its own characteristics, including beliefs, traditions, and gender-related specifications, which should be taken into consideration, to offer suitable, relatable solutions and avoid inappropriate content.

#### Religious Considerations

When creating CMH solutions specifically for Emiratis or Arabs in general, including religious content might offer a sense of trust and comfort to many users. However, as the United Arab Emirates has a mixed population of people with different religious backgrounds, religion should be respected in the sense of avoiding the inclusion of any shapes, images, illustrations, or colors that might have religious meanings and could offend certain users.

#### Users’ Health and Data Safety

#### Confidentiality

Health information in general is sensitive, especially mental health information. Data privacy and security should be a top priority when designing CMH interventions to protect the users; if patients’ data fall into the wrong hands, it could be used to harm them [53]. Patients should be assured that their data would not be hacked or leaked to encourage them to use the CMH interventions and share their data.

#### Respecting Legal and Ethical Guidelines

Law and ethics for psychological treatment in general and for use of technology differ from one country to another. Existing laws and ethics should be investigated and respected. There are laws in the United Arab Emirates regarding use of technology and data disclosure. For example, Article 379 of the UAE Penal Code prohibits a person, who by means of their profession is entrusted with a *secret*, from disclosing that *secret* or information without consent [54]. In addition, Federal Law No. 5 of 2012 and its amendment Federal Law No. 12 of 2016 prohibit individuals from using any electronic information systems or any information technology tools to offend or invade another person’s privacy without authorization [55,56].

#### Users’ Suitability Assessment

CMH suitability testing is imperative to avoid putting any user at risk. Testing could be either conducted by an MHP or based on validated psychological tests such as the Depression Anxiety and Stress Scale [57,58]. Psychological testing would help acquire an overview on the psychological state of the user to determine whether the intervention could be beneficial and appropriate for the specific user.

#### Risk and Crisis Management Features

Some users might go through crises and situations that may require immediate intervention from MHPs or family members. Features to manage these situations should be considered to ensure the safety of users. Such features include automatic messages to family or caregivers, providing hotlines’ contact, and providing directions that could help the user overcome the crisis or risk situation.

#### Ensuring MHP Integrity and Content Safety

CMH interventions should ensure that any content or input presenting mental care treatments, advice, or practices is sourced from legitimate and licensed MHPs. Illegitimate or harmful input can be a major issue when the CMH solution provides features such as chat between users, web-based communication between the community of the interventions’ users, posts, comments, or any features that enable insertion of an input. All these features should imperatively be monitored by an MHP to eliminate harmful content.

#### Technical Considerations

#### Good and Clear Communication

Good and clear communication between the patient and the MHP or caregiver is crucial. Mental health is a sensitive subject, and the patient should be put in an environment without interruptions and difficulties, such as unclear video communication or sound. The United Arab Emirates offers good-quality internet network, with one of the fastest data and download speeds in the world [59,60]. CMH interventions should take advantage of the UAE network quality and ensure that technical issues that would interrupt the communication between the patient and the MHP are avoided.

#### Providing Technical Support and Technology Education to Both Patients and MHPs

Some MHPs and patients may not be accustomed to or comfortable using technology; therefore, technology education and training may be necessary before use. In addition, technical support should be available in case of need.

#### Availability of Solutions in Formats Compatible With Mobile Phones

Mobile phones are one of the most used devices. Availability of the CMH solutions in formats that could be used and accessed through mobile phones could help increase the accessibility to CMH solutions and promote their use.

### Limitations

This study may have some limitations: (1) the number of MHPs who responded to the study was low, mainly because of the timing of the investigation being during the COVID-19 lockdown, which made reaching a large number of participants difficult, and (2) conducting interviews with the participants would have provided valuable insights, but because of the timing of the study, conducting face-to-face interviews was not possible.

### Conclusions

CMH interventions could help overcome many of the existing mental care delivery barriers in the United Arab Emirates, mainly stigma and the availability of MHPs. Surveyed MHPs confirmed the utility and possible adoption of CMH in the United Arab Emirates and provided a number of factors that should be considered when creating CMH solutions for the UAE population. These factors mainly included cultural, religious, and linguistic considerations. However, MHPs also expressed certain concerns around the importance of physician–patient relationship, which could be jeopardized with patients’ reliance on CMH for mental care, in addition to patients’ suitability concerns, as CMH interventions may not be suitable and beneficial for all patients. On the basis of the MHP input, a blended care approach, combining both traditional treatment and CMH solutions, was concluded to be potentially suitable, beneficial, and successful in the UAE context.

Surveyed MHPs have also reported a set of concerns regarding CMH adoption and a set of critical features that should exist in CMH solutions. On the basis of the input of MHPs, a list of design features to consider when creating CMH interventions for the United Arab Emirates was presented.

## Abbreviations

CMH: connected mental health
ICT: information and communication technology
PRISMA: Preferred Reporting Items for Systematic Reviews and Meta-Analyses
